# Role of seed infection for the near and far distance dissemination of wheat blast caused by *Magnaporthe oryzae* pathotype *Triticum*

**DOI:** 10.3389/fmicb.2023.1040605

**Published:** 2023-02-01

**Authors:** Musrat Zahan Surovy, Tofazzal Islam, Andreas von Tiedemann

**Affiliations:** ^1^Division of Plant Pathology and Crop Protection, Department of Crop Sciences, Georg-August-Universität Göttingen, Göttingen, Germany; ^2^Institute of Biotechnology and Genetic Engineering, Bangabandhu Sheikh Mujibur Rahman Agricultural University, Gazipur, Bangladesh

**Keywords:** MoT, epidemiology, seed germ, seed transmission, healthy seeds

## Abstract

*Magnaporthe oryzae* pathotype *Triticum* (MoT) is a devastating fungal phytopathogen causing wheat blast disease which threatens wheat production particularly in warmer climate zones. Effective disease control is hampered by the limited knowledge on the life cycle, epidemiology, and pathogenicity of MoT. Since MoT mainly infects and colonizes the inflorescences of wheat, infection, invasion routes and colonization of MoT on wheat ears and in wheat seeds were investigated in order to assess potential seed transmission pathways. MoT was spray inoculated on two wheat cultivars (Sumai 3, susceptible and Milan, resistant) at three ear maturity stages [full ear emergence, growth stage (GS) 59; mid flowering, GS 65; and end of flowering, GS 69]. Incidence of MoT on Sumai 3 seeds was 100% and 20–25% on Milan. MoT sporulation rate on Sumai 3 contaminated seeds was more than 15 times higher than on Milan. Repeated washes of seed samples for removing paraffin fixation hampers seed microscopy. To overcome the damage of seed samples, we used hand-sectioned seed samples instead of paraffin-fixed microtome samples to facilitate microscopy. The colonization of MoT within various seed tissues was followed by light and confocal laser scanning microscopy (CLSM). Invasion of MoT in seeds predominantly occurred in the caryopsis germ region, but entry *via* other seed parts was also observed, confirming the potential of intense colonization of MoT in wheat grains. Fungal spread in wheat plants growing from MoT infected seeds was monitored through plating, microscopic and molecular techniques. Under greenhouse conditions, no spread of MoT from infected seeds to seedlings later than GS 21 or to ears was detected, neither in Milan nor in Sumai 3. We therefore conclude, that MoT may not systemically contaminate inflorescences and seeds in neither susceptible nor resistant wheat cultivars. However, initial blast symptoms, only found on seedlings of Sumai 3 but not Milan, resulted in the formation of new conidia, which may serve as inoculum source for plant-to-plant dissemination by airborne infection of plant stands in the field (short distance spread). Ultimately the inoculum may infect young inflorescences in the field and contaminate seeds. Our findings again stress the risk of long-distance dissemination of wheat blast across continents through MoT-contaminated seeds. This underlines the importance of mandatory use of healthy seeds in strategies to control any further spread of wheat blast.

**Graphical Abstract:**
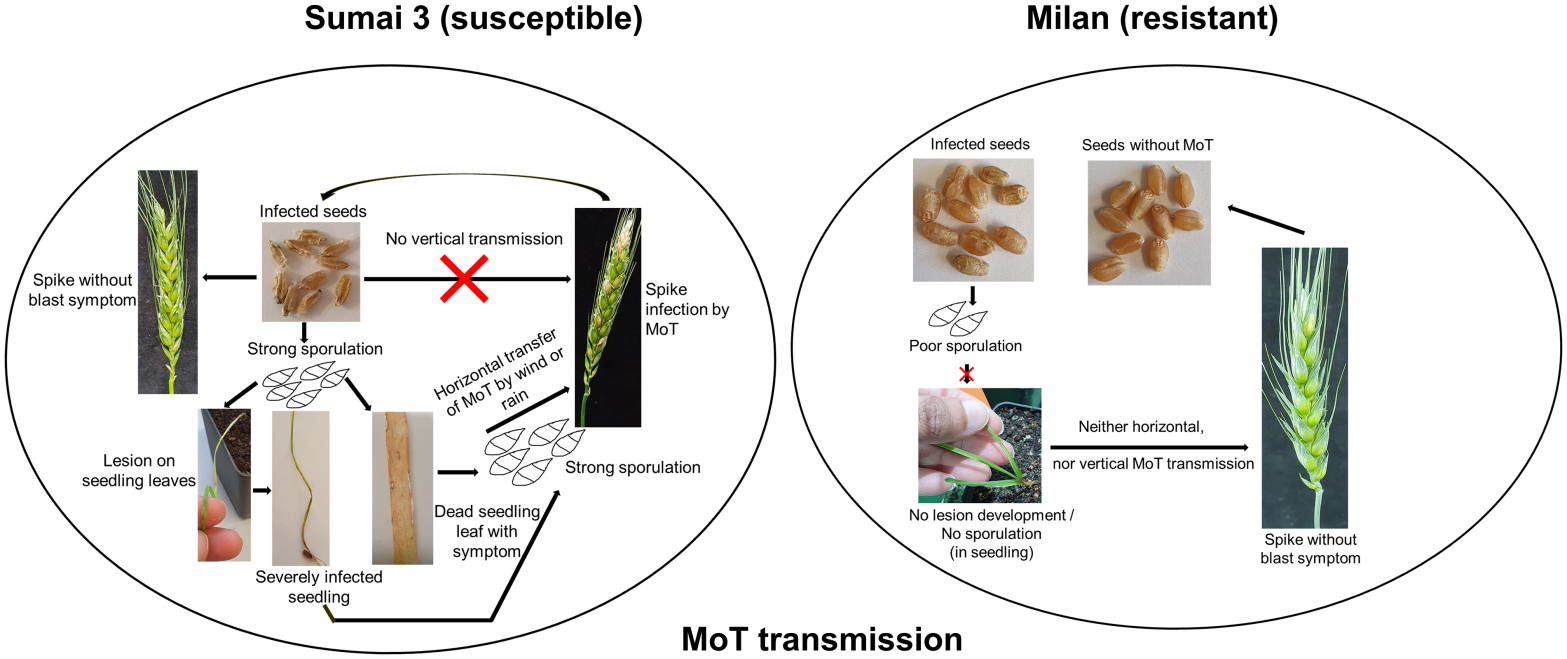


## 1. Introduction

Seeds are a main source of inoculum and the potential starting point of numerous diseases (Elmer, 2001). Besides seed-to-plant transmission, either by systemic infection of seedlings or by (horizontal) infection of neighbor plants by seed-borne spores, there also exists a (vertical) seed-to-seed transmission, which allows long-range spread of pathogens through seed trade (Baker and Smith, 1966; Shahzad et al., 2018; Nallathambi et al., 2021). The latter may lead to the introduction and establishment of a pathogen in a new region (Elmer, 2001). Hence, the potential role of seeds in spreading plant diseases is essential for understanding plant disease epidemiology and for assessing the risk of long distance dissemination (McGee, 1979).

Wheat is an important cereal food crop and grown on more than 200 million hectares of cropland worldwide (Tesfaye, 2021). It can grow under a wide range of climatic conditions (Shiferaw et al., 2013) and provides 20% of global dietary protein and calories (FAO, 2015), thus playing a crucial role in global food security. Fungal pathogens may threaten wheat production (Stokstad, 2007; Winston, 2015). The hemibiotrophic fungus *Magnaporthe oryzae* (anamorph *Pyricularia oryzae*) pathotype *Triticum* (MoT) causes wheat blast, a disease which may reduce yield up to 100% in susceptible cultivars (Islam et al., 2016, 2020). This disease first occurred in Brazil (Igarashi et al., 1985), then spread to Bolivia, Argentina, Paraguay, and more recently to Bangladesh and Zambia (Ceresini et al., 2019; Tembo et al., 2020; Singh et al., 2021). In 2016, Bangladesh imported a large amount of wheat from South America, which is supposed to have introduced wheat blast to Bangladesh (Ceresini et al., 2018; Mottaleb et al., 2018). Warm and humid weather is conducive for wheat blast epidemics as experienced by the extraordinary warm and humid weather conditions in 2016 which facilitated the wheat blast outbreak in Bangladesh (Islam et al., 2019).

The wheat blast pathogen MoT may infect leaves and stems, but ear infection is clearly prevailing in the field, whereas leaf symptoms are rarely observed (Urashima et al., 2004). Basal senescent wheat leaves may however produce conidia and play a role in the secondary distribution of wheat blast in the field (Cruz et al., 2015). Blast conidia may survive for 5 months in crop residues, like stems, rachis and leaves (Pizolotto et al., 2019) and for 3 years in rice stems and straw (Raveloson et al., 2018). In wheat seeds, MoT can survive for 19–22 months (Reis, 1995) maintaining a viability of 60% (Maciel, 2018). MoT inoculum is mainly dispersed by rain, and wind, but seeds have also been reported to play a role (Urashima et al., 2007; Islam et al., 2019). Barley (*Hordeum vulgare*), maize (*Zea mays*), oat (*Avena sativa*), swamp grass (*Leersia hexandra*), and goosegrass (*Eleusine indica L*.) are alternative hosts of MoT (Maciel et al., 2014; Cruz and Valent, 2017; Gupta et al., 2021). Conversely, the rice blast pathogen *Magnaporthe oryzae* pathotype *oryzae* (MoO) has been reported to occasionally infect wheat (Shizhen et al., 2021; Paul et al., 2022), while rice does not normally serve as a host for MoT (Monsur et al., 2016). However, Tingting (2014) reported that some MoT strains may also infect rice seedlings.

Ear infection results in deformed, shriveled, and discolored grains (Urashima et al., 2009; Cruz and Valent, 2017; Martínez et al., 2019; Surovy et al., 2020). It was reported that wheat blast infection deteriorated the grain quality by modulating the grain protein content (Surovy et al., 2020). Data from previous studies indicate that MoT infected seeds may cause more than 20% disease incidence in the offspring (Goulart et al., 1990; Goulart and Paiva, 2000). However, 42 days after sowing, MoT could not be isolated from wheat plants grown from infected seeds (Martinez et al., 2021). Taking previous reports together, the mode of disease transfer by seeds infected with MoT is not clear. There are two possible ways of seed transmission of MoT, (i) by superficial attachment on the seed surface and transmission by wind, rain, or other mechanical ways into the field (horizontal transfer), or (ii) by seeds internally producing systemically infected plants and MoT-infected grains (vertical transmission).

The detection of MoT from superficially or internally colonized seeds is possible through plating and microscopic techniques, but the symptomless internal colonization is quite difficult to detect only by these methods. Proper localization of MoT in the seeds, however, is crucial to understand the systemic transmission of MoT from seeds to plants or next-generation seeds. Previous studies investigating transmission of blast pathogens (Faivre-Rampant et al., 2013; Martinez et al., 2021) used visual identification followed by plating and fluorescence microscopy. Until today, a comprehensive study addressing the potential of vertical seed-to-seed transmission of MoT in wheat and the localization of MoT in seeds is lacking. The present study combined plating, microscopy and quantitative polymerase chain reaction (qPCR) in order to address the following questions, (i) which parts of the seeds are preferentially colonized by MoT, (ii) is MoT transmitted vertically in a symptomless manner to infect next generation seeds, and (iii) how can infected seeds produce diseased plants with ear blast symptoms? The overall aim of this study was to investigate the role of infected wheat seeds in the dissemination of blast disease within a field (short distance) and from far away regions (long distance).

## 2. Materials and methods

### 2.1. Plant material

Two wheat cultivars, susceptible Sumai 3 and resistant Milan [CIMMYT wheat advanced line carrying 2NS/2AS translocation (Téllez et al., 2022)] were selected. Seeds were surface sterilized with 3% NaOCl for 1 min and subsequently washed three times with sterilized distilled water (SDW). Surface-sterilized seeds were kept in 90 mm Petri dishes for 48 h in humid conditions to facilitate germination. Pre-germinated seeds were sown in plastic pots (two per pot; 9 cm× 9 cm× 9.5 cm) filled with a mixture of compost, sand, and peat [2:1:1, volume basis (v/v/v)]. The plants were grown in a greenhouse at 14 h daylight, 25°C (±1), and 65–70% relative humidity (RH). Bottom watering was performed based on water demand. Hakaphos blue nutrient solution (3 g/l, COMPO, Muenster, Germany) was applied weekly beginning at GS 20 (tillering stage, Zadoks et al., 1974). One tiller per plant and two primary tillers in each pot were maintained.

### 2.2. Fungal isolate

Wheat blast isolate BTGP-6f was obtained through single conidia isolation method from the infected ears collected from a blast affected farmer’s field in Meherpur of Bangladesh by the Institute of Biotechnology and Genetic Engineering (IBGE), Bangabandhu Sheikh Mujibur Rahman Agricultural University, Bangladesh (Gupta et al., 2020). The fungal isolate was cultured on 90 mm Petri dishes containing 5% V8 agar medium (50 ml V8 juice, 2 g CaCO_3_, 15 g Agar, 950 ml water) supplemented with streptomycin (200 ppm) and incubated in a growth chamber (SANYO growth cabinet MLR 350, EWALD Innovationstechnik GmbH, Germany) at 25°C (±1) under continuous fluorescent light (fluorescent lamp 40 W, 20,000 lumens). The conidial suspension was prepared from 7-days old MoT cultures by adding 10 ml of 0.01% sterile Tween 20 aqueous solution per plate. The conidial density was adjusted to 1 × 10^5^ conidia/ml using a Neubauer hemocytometer (Fuchs-Rosenthal, 0.0625 mm^2^) (Surovy et al., 2022).

### 2.3. Inoculation procedure

Uniform ears were artificially inoculated with MoT (1 × 10^5^ conidia/ml) at three different growth stages (GS 59, full ear emergence; GS 65, mid flowering; and GS 69, end of flowering) (Zadoks et al., 1974). An air compressor (Mini air compressor K17, Type N022 An. 18) was used for spray inoculation, and *ca.* 2 ml of conidial suspensions were sprayed per ear while distilled water was used as control (Ha et al., 2016). Twelve pots for each inoculation time point and each cultivar were prepared. Humidity was immediately maintained by covering inoculated ears with plastic bags. Plastic bags were removed 24 h after inoculation, and plants were kept in a climate chamber with a 12 h/12 h day-night photoperiod with 120 μmol m^−2^ s^−1^ (±5) light intensity (high-pressure sodium lamp) at 25°C (±1). Wheat plants were organized in a completely randomized design (CRD), and the ear samples were collected at GS 91 (grain hard). Two repetitive experiments were conducted.

### 2.4. Assessment of seed germination and MoT infection rate

Thirty randomly selected surface-sterilized seeds (single replicate) were placed in a 90 mm Petri dish containing PDA (4 g potato extract, 20 g dextrose, 15 g agar, 1,000 ml water, pH 5.6) to determine percentage of seed infection by MoT. Three replicates for each inoculation time point of each cultivar were assessed. Visual and microscopic observation for the presence of MoT conidia in seeds or grown mycelia were conducted to determine the MoT seed infection rate. The percentage seed infection was calculated 5 days after plating using the formula used by Surovy et al. (2020):


$$\mathrm{Seed infection}\;\left(\%\right)=\left(\frac{\mathrm{Number of seeds infected}}{\mathrm{Total number of seeds used}\;}\right)\times 100\%$$

For assessment of seed germination, 25 randomly selected surface-sterilized seeds (single replication) from each inoculation time point of each cultivar were placed in a Petri dish containing sterilized distilled water-soaked filter paper (90 mm, Ref# 41255009, Glaswarenfabrik Karl Hecht GmBH & Co KG, Sondheim, Germany) for germination assay. Three replications were maintained. The percentage of germination was calculated 7 days after incubation following the formula used by Surovy et al. (2020):


Seed germination(%)=(Number of seeds germinatedTotal number of seeds used for germination)×100%

### 2.5. Assessment of sporulation on seeds

Twenty seeds per replication were randomly selected from each inoculation time point of each cultivar for the MoT sporulation assay. Three replicates were prepared for each treatment. Surface sterilized seeds were placed in sterile micro-slides (76 × 26 mm, Glaswarenfabrik Karl Hecht GmbH and Co KG, Germany) in a 90 mm Petri dish (single seed placed in single slide) containing sterilized distilled water-soaked sterile filter paper to maintain humidity. Twenty-four to 48 h after incubation, seeds were observed under a stereomicroscope (Leica Wild M10) followed by a compound light microscope (Leica Leitz DM RB). The samples were photographed with a Leica DFC420 camera (version 2.8.0.0), and all images were processed using Leica Application Suite V4 (LAS V4) software (version 4.9.0.129). Subsequently, a single infected seed presenting MoT conidia was placed in a 1.5 ml Eppendorf tube containing 1 ml of SDW and vortexed briefly. After vortexing, 5 μl of suspension was poured into each counting chamber of hemocytometer (Fuchs-Rosenthal, 0.0625 mm^2^) to check the homogeneity of the conidial suspension before the conidia were counted. This procedure was repeated five times, and the average counts were multiplied by 200 to obtain the total number of conidia per seed.

### 2.6. Assessment of seed-to-seed transmission of MoT

Visually infected seeds from artificial inoculation assays were further used in greenhouse experiments to investigate seed-to-seed transmission of MoT. A composite mixed sample of seeds collected from all three inoculation time points was prepared from Sumai 3 and Milan. Randomly selected seeds from the composite mixture were washed with SDW and pre-germinated in 90 mm Petri dishes. Pre-germinated seeds were sown in plastic pots (one per pot; 7 cm × 7 cm × 8 cm) containing similar potting mixture described previously (see above 2.1). Each pot was treated as a replicate, and 60 replicates were used for each cultivar. Wheat plants were grown in a climate chamber, maintaining same conditions as described above (section 2.3). Seedlings were subjected to careful daily observation for disease symptoms, and the experiment was repeated once. Samples were taken for further analysis from seedling leaves, stems, and ears at GS 11, GS 14, GS 21, GS 55, GS 65, and GS 91.

### 2.7. Reisolation of conidia from the offspring of MoT-infected seeds

Plant samples were surface sterilized with 3% NaOCl and placed in a humid chamber for 24–48 h at 25°C (±1) for germination of MoT conidia. The presence of characteristic three–celled pyriform MoT conidia was examined under stereo and compound microscopes and also plated on synthetic nutrient-poor agar (SNA) (1 g KNO_3_, 1 g KH_2_PO_4_, 0.5 g MgSO_4_ × 7H_2_O, 0.5 g KCl, 0.2 g glucose, 0.2 g sucrose, 20 g agar, 1,000 ml H_2_O) supplemented with streptomycin (200 ppm). Sporulation of MoT on SNA was again checked for further confirmation of MoT from plant samples.

### 2.8. Localization of MoT in infected seeds and plant samples by confocal laser scanning microscopy (CLSM)

Seed caryopsis samples for cytological analyses were collected from Sumai 3 and Milan ears at 10 and 14 days after MoT inoculation on ears. Seed samples were stored in ACE solution (25% ethanol: 75% chloroform, adjusted to pH 2.8 with 0.15% trichloroacetic acid). Ten to fifteen caryopses were transversely cross-sectioned by hand for microscopic analysis (30–40 sections from each inoculation time point of each cultivar).

Infected leaves and stems (at least six) of wheat plants derived from MoT contaminated seeds were also stored in ACE solution for further analysis. The leaf samples were cut into small pieces, whereas stems and seeds were cross-sectioned using razor blades (Wilkinson sword, Germany), and all samples were double-stained with Wheat Germ Agglutinin Alexa Fluor 488 conjugate, AF (ThermoFisher SCIENTIFIC, Catalogue number W11261), and propidium iodide, PI (SIGMA, Catalogue number P4170-10MG). For leaf and stem samples, AF was added (30 μl of 50 μg/ml) and vacuum infiltrated for 20 min. Later, PI was added (30 μl of 10 μg/ml) and again vacuum infiltrated for 10 min. Vacuum infiltration was done at 250 millibars (mbs) with a regular interval of every 5 min (Ha et al., 2016). The AF staining of seed samples was done for 2 h and PI for 30 min. After staining, the specimens were mounted immediately in 50% glycerol for observation or stored dark at 4°C for further analysis. The CLSM was carried out with a Leica TCS SP5 laser scanning microscope 510 (Leica Microsystems CMS GmbH, Germany). AF was excited using a 488 nm laser and collected at 500–540 nm. The fluorescence signal of PI was excited at 514, and emission recorded at 580–660 nm. CLSM images were processed using Leica Application Suite Advanced Fluorescence software (LAS AF), Version 2.6.3.8173.

### 2.9. Detection of MoT in plant samples by qPCR

Asymptomatic wheat seedlings and ear samples from Milan at different growth stages (GS 11, GS 14, GS 21, GS 55, GS 65, and GS 91) and ear samples from Sumai 3 collected at GS 55, GS 65, and GS 91 were used for qPCR analysis. At each time point, six ear samples were randomly selected for qPCR analysis for each cultivar.

Collected fresh samples were immediately frozen in liquid nitrogen and ground in a ball mixer mill (Mixer Mill MM400, Retsch). The DNA was extracted by the modified cetyltrimethylammonium bromide (CTAB) method with polyethylene glycol (PEG) precipitation (Ha et al., 2016; Alisaac et al., 2020). The extracted DNA was dissolved in 100 μl TE buffer (10 mM Tris, 1 mM EDTA, pH 8.0). The quality of DNA was checked in a 1.0% agarose gel electrophoresis. DNA concentration was measured using a microplate spectrophotometer (Epoch, Bio-tech) at 260 nm and controlled by the ratio of OD260/OD280. The target sequence was quantified by a thermal cycler (BioRad C1000 Touch™ Thermal Cycler, CFX384™Real-Time System), maintaining a standard curve of pure MoT DNA with 10-fold dilutions (from 1.0 ng to 0.01 pg./μl). The qPCR data were further analyzed with BioRad CFX Maestro 1.1 (Bio-Rad) software. The presence of MoT DNA was determined by species-specific primers [forward primer, *pfh2a* (5 ´-CGTCACACGTTCTTCAACC-3 ´) and reverse primer *pfh2b* (5 ´-CGTTTCACGCTTCTCCG-3 ´), synthesized by InvitrogenTM (Thermo Fisher Scientific)]. Briefly, the PCR was performed with an initial denaturation at 95°C for 3 min, followed by denaturation at 95°C for 15 s for 35 cycles, annealing at 58°C for 25 s, and elongation at 72°C for 45 s. The final elongation was carried out for 2 min at 72°C. Three technical replicates were performed for each biological replicate.

### 2.10. Statistical data analysis

All experimental data were analyzed by R software (version 4.0.5, accessed 31 March 2021) integrated with R studio (version 1.2.5001, accessed 31 March 2021). Linear models (LMs) and generalized linear models (GLMs) were used to analyze the relationship between explanatory variables (% seed infection, % reduction of seed germination, number of conidia per seed) and the effects of MoT on wheat cultivars. The model’s dispersion and residuals were tested using the function test ‘dispersion’ and ‘stimulatedResiduals’ of the “DHARMa” package. These functions are a simulation-based approach to create readily interpretable scaled residuals for the fitted linear models. Seed infection (%) and seed germination (%) were analyzed by using linear models (ANOVA), and the number of conidia per seed was analyzed by generalized linear models (GLMs) with negative binomial (link = log) family. For multiple comparisons “emmeans” package was used with “Tukey” function. The Spearman Rank correlation was performed to determine the relationship between seed infection percentage and reduction of seed germination percentage. For visualization of bar graphs, “ggplot2” package was used and to combine two or more graphs, “gridextra” package was used. The “ggpubr” function was used to visualize the Spearman Rank correlation data, and the staked graph was prepared using the Microsoft office excel program (Microsoft Office Professional Plus 2016, version 16.0.4266.1001).

## 3. Results

### 3.1. Effect of MoT inoculation on seed infection percentage

*Magnaporthe oryzae* pathotype *Triticum*-inoculated ears of susceptible Sumai 3 and resistant Milan were collected at GS 91 to determine percentage of seed infection by MoT. There were no significant interactions between cultivar and inoculation time points on MoT seed infection percentage (*F* = 0.09, *p* = 0.914). In all three inoculation time points, seed infection was significantly higher in Sumai 3 than in Milan (*F* = 325.20, *p* < 0.001), while no significant effects were found for different inoculation time points (*F* = 1.022, *p* = 0.389) (Table 1).

**Table 1:** ANOVA for the effect of MoT on seed infection percentage.

Variables	df	*F* value	*p-*value
Cultivar (C)	1	325.20	<0.001
Inoculation time points (ITPs)	2	1.022	0.389
C: ITPs	2	0.09	0.914

**Figure 1:**
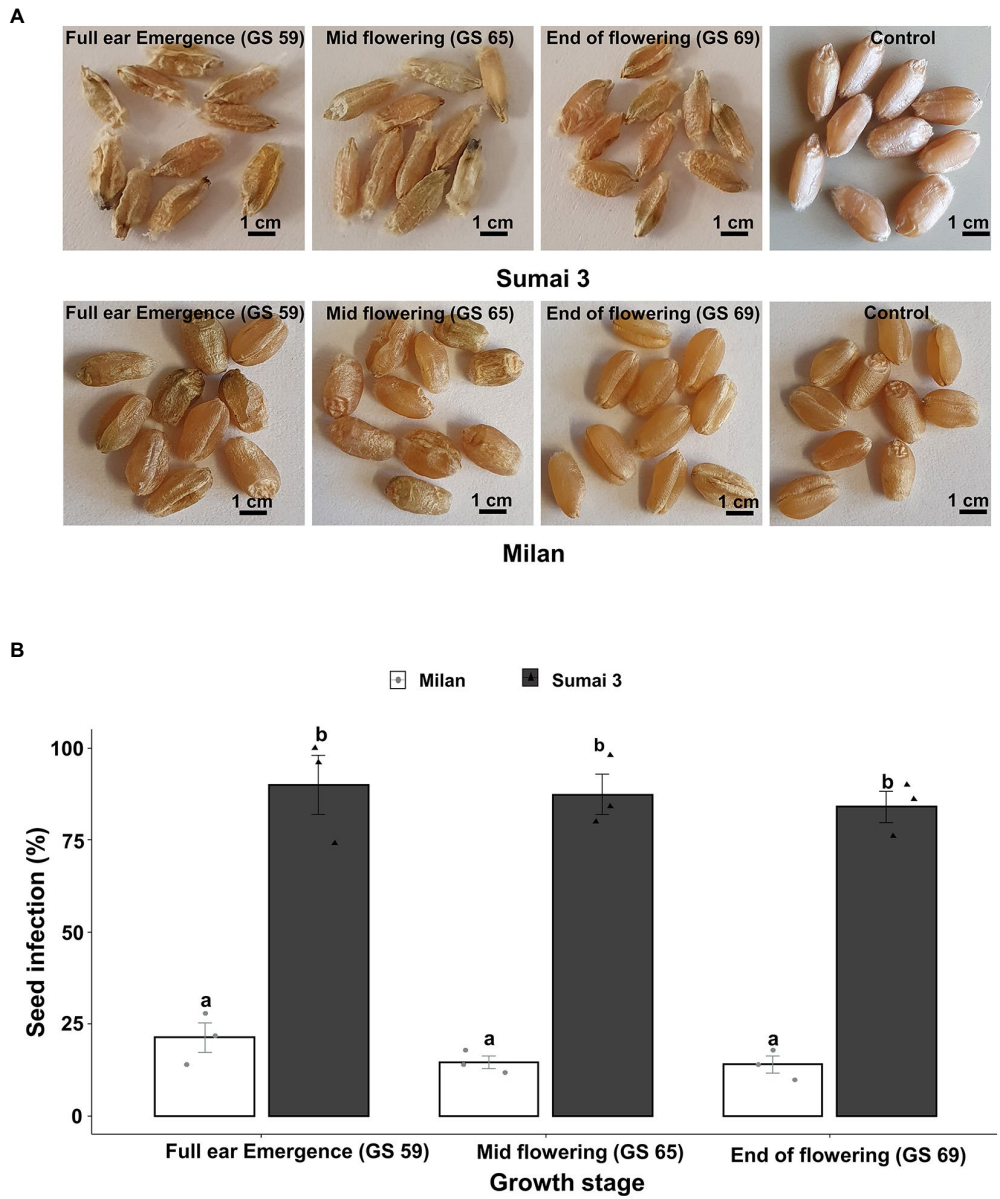
Effect of MoT inoculation of wheat ears on MoT seed infection of Sumai 3 and Milan in greenhouse conditions. **(A)** Mature seeds of Sumai 3 and Milan were collected from ears inoculated at three different ear maturity stages (GS 59, full ear emergence; GS 65, mid flowering; and GS 69, end of flowering); **(B)** Seed infection with MoT in Sumai 3 and Milan cultivars assessed by plating assay under greenhouse conditions. Surface sterilized seeds were incubated on PDA at 25°C for 5 days. The mean (±standard error) of seed infection (%) of each ear inoculation time point is represented by each bar. ANOVA with Tukey test was performed, and treatments with the same letters at the cultivar level were not significantly different (*n* = 3; one replication contained 30 seeds of each cultivar at each inoculation time point; *p* = 0.05). Each data point represents one replicate consisting of 30 randomly selected seeds.

Seed phenotyping from the greenhouse experiment confirmed that, Sumai 3 was more susceptible to MoT, showing severely deformed, shriveled, and blackish grains, than Milan (Figure 1A). In both cultivars, no disease symptoms were recorded in mock-inoculated control seeds. Seed infection in Sumai 3 ranged from 74 to 100%, while in Milan, it ranged from 10 to 28% (Figures 1A,B).

### 3.2. Effect of MoT inoculation on seed germination

**Figure 2:**
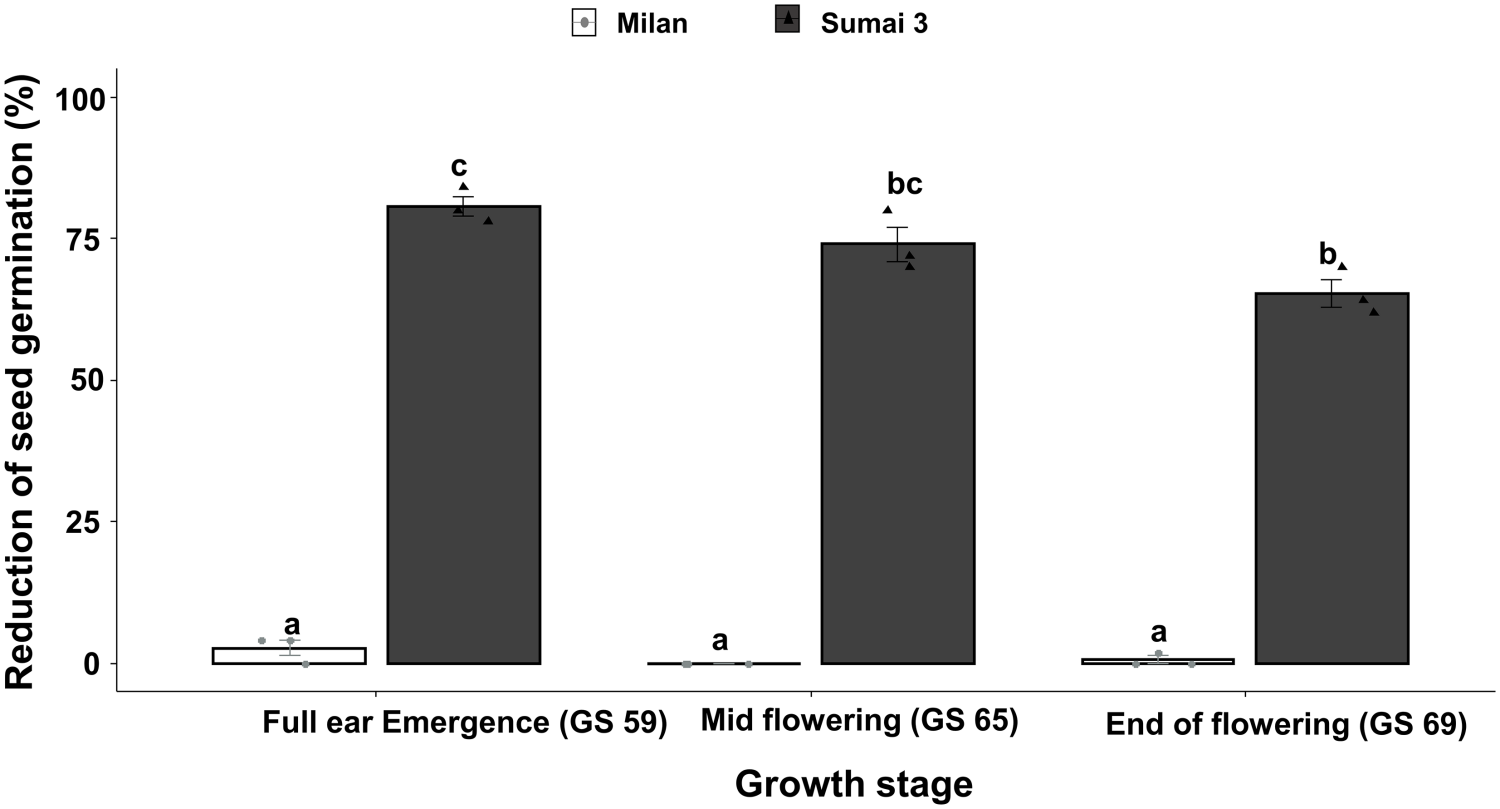
Effect of different MoT inoculation time points on the reduction of (%) seed germination of Sumai 3 and Milan in greenhouse conditions. The wheat ears were inoculated with a conidial suspension of 1 × 10^5^ conidia/ml at GS 59, full ear emergence; GS 65, mid flowering; and GS 69, end of flowering stages. For each cultivar, the mean (± standard error) of reduction of seed germination (%) of each ear inoculation time point is represented by a bar. ANOVA with Tukey test was performed, and treatments with the same letters at the cultivar level were not significantly different (*n* = 3; one replication contained 25 seeds of each cultivar at each inoculation time point; *p* = 0.05). Each data point represents one replicate consisting of 25 randomly selected seeds.

Parallel to the seed infection assessment, seeds collected at GS 91 were used for seed germination assay. Cultivars (*p* < 0.001), inoculation time points (*p* = 0.001) and their interactions (*p* = 0.01) had statistically significant effects on seed germination percentage. In all three inoculation time points, the seed germination rate of Sumai 3 was significantly lower than in Milan, varying from 80.7% on ears inoculated at GS 59, to 65.3% on ears inoculated at GS 69 (Figure 2). In contrast, there was no statistically significant difference in the reduction of seed germination at different inoculation time points for Milan.

### 3.3. Correlation between seed infection by MoT and seed germination

**Figure 3:**
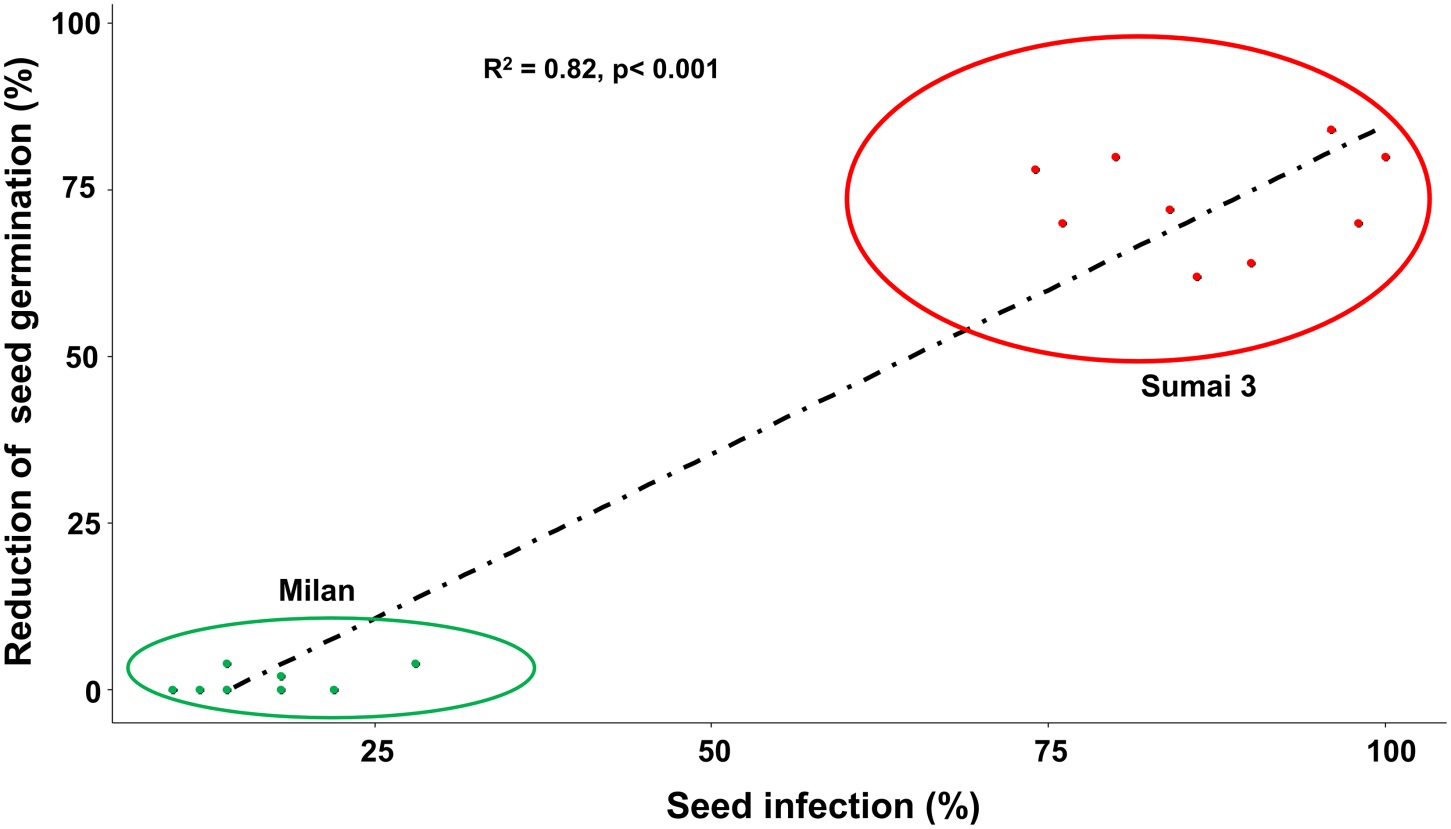
Relationship between MoT seed infection (%) and reduction of seed germination (%) of Sumai 3 (red) and Milan (green). Spearman Rank correlation analysis was performed to visualize the correlation data.

Based on Spearman Rank correlation analysis, seed infection by MoT was positively related to the reduction of seed germination percentage. Higher MoT infection in seeds resulted in less germination rates of seeds (*R* = 82%), and these interactions were significantly different (*p* < 0.001) between cultivars (Figure 3).

### 3.4. Sporulation of MoT conidia from MoT-infected seeds

**Figure 4:**
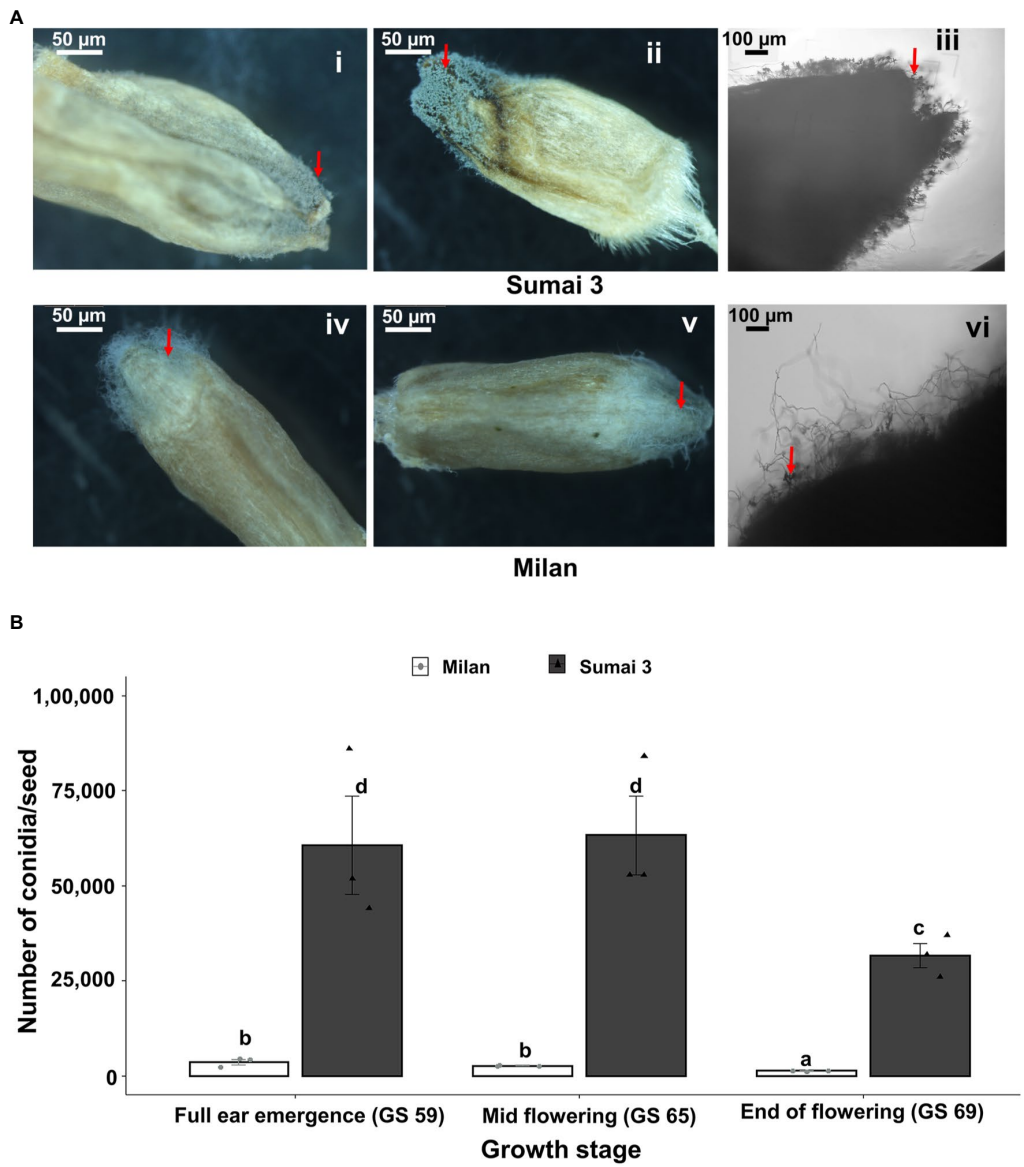
Sporulation rate of MoT in seeds from wheat plants spray inoculated with a MoT conidial suspension of 1 × 10^5^ conidia/ml on ears at three different flowering stages. **(A)** MoT conidia on representative seeds of Sumai 3 and Milan. The red arrow indicates the sporulation of MoT conidia on the seeds. **Ai**, **Aii**, **Aiv**, **Av** (scale bar 50 μm) were photographed under a stereomicroscope, **Aiii** and **Avi** were photographed under a compound light microscope (scale bar 100 μm). **(B)** Number of MoT conidia per seed in Sumai 3 and Milan seeds collected from different inoculation time points. For each cultivar, bars represent the mean (± standard error) of total number of conidia per seed of each ear inoculation time point. ANOVA with Tukey test was performed, and treatments with the same letters at the cultivar level were not significantly different (*n* = 3; one replicate contained 20 seeds of each cultivar at each inoculation time point; *p* = 0.05). Each data point represents one replicate consisting of 20 randomly selected seeds.

Sporulation of MoT was detected on seeds of both Sumai 3 and Milan collected from all inoculation time points (full ear emergence, GS 59; mid flowering, GS 65; and end of flowering stages, GS 69). The highest sporulation rate was consistently found in Sumai 3 which significantly differed from Milan (*p* < 0.001). Upon close inspection of seeds under a stereomicroscope, more MoT sporulation was noticed in the germ region than in other seed parts in both cultivars (Figure 4A).

In the susceptible cultivar Sumai 3, the number of conidia per seed ranged from 31,667 to 63,334. Number of conidia per seed did not significantly differ between ears inoculated at GS 65 (63,334 conidia/seed) and GS 59 (60,666 conidia/seed), however, the lowest number of conidia was found in seeds collected from GS 69 (31,667 conidia/seed), which was significantly different from GS 59 and GS 65.

In Milan, the number of conidia per seed ranged from 1,354 to 3,688. Like Sumai 3, the lowest number of conidia was recorded in seeds inoculated at GS 69 (1,354 conidia/seed), which significantly differed from GS 59 (3,688 conidia/seed) and GS 65 (2,729 conidia per seed) (Figure 4B).

### 3.5. Detection of MoT in seeds from inoculated ears through CLSM

**Figure 5:**
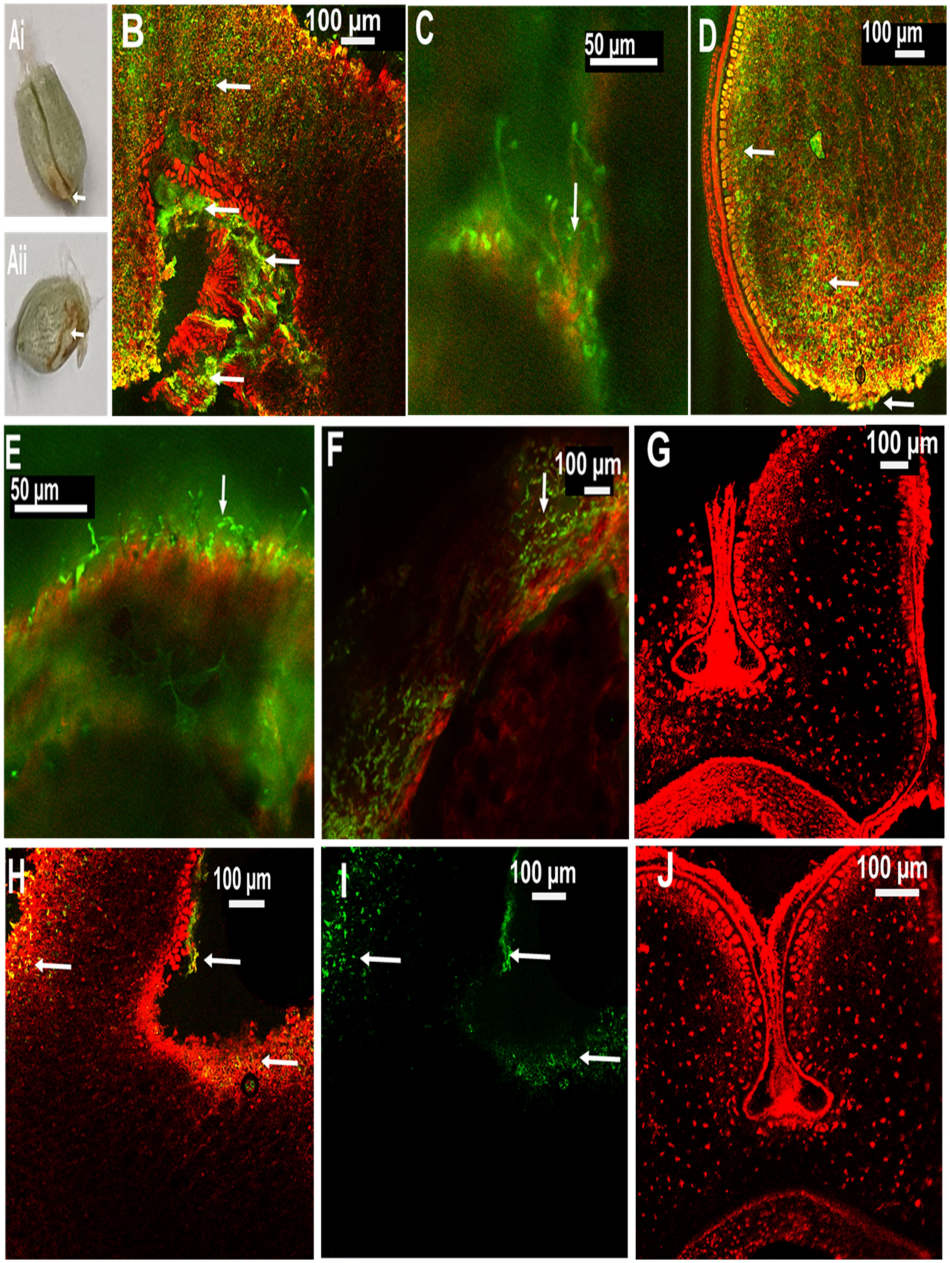
Localization of MoT on wheat seeds by confocal laser scanning microscopy (CLSM) using Alexa Flour 488 (AF), staining fungal material green and Propidium Iodide (PI) staining plant tissue red. **(A)** Seeds of wheat cv. Sumai 3 infected with MoT, where **(Ai)** inoculated at mid flowering stage, GS 65 and sampled at 10 dpi and **(Aii)** inoculated at the end of flowering stage, GS 69 and sampled at 10 dpi. **(B)** Overlay image of a cross-section of the germ region of an infected Sumai 3 seed (inoculated at GS 65 and sampled at 14 dpi). **(C)** MoT hyphae in germ endosperm (enlarged view from **B**). **(D)** Cross-section of MoT infected caryopsis of Sumai 3 (infected at seed coat region) (inoculated at GS 69 and sampled at 10 dpi); **(E)** MoT hyphae in the caryopsis coat tissue of Sumai 3 (inoculated at GS 69 and sampled at 10 dpi). **(F)** MoT hyphae within seed pericarp and testa region (enlarged view from **B**). **(G)** Cross-section of Sumai 3 healthy caryopsis. **(H)** Overlay image of a cross-section of the germ region of infected Milan seed (inoculated at GS 65 and sampled at 14 dpi). **(I)** MoT hyphae in infected Milan caryopsis germ region-single channel excitation (inoculated at GS 65 and sampled at 14 dpi). **(J)** Cross-section of a healthy caryopsis of Milan. The scale bar of images **(B,C,E)** is 50 μm, and the rest is 100 μm. White arrows indicate presence of MoT hyphae.

In order to localize MoT in seeds from inoculated ears, CLSM microscopy was performed with seed sections. The internal tissues of the healthy caryopses of Sumai 3 and Milan were entirely free of MoT hyphae (Figures 5G,J). However, after MoT inoculation, the caryopsis pericarp was severely damaged and deformed in Sumai 3. Massive hyphal growth was detected in the caryopsis coat tissues and endosperm of Sumai 3 seeds. When MoT hyphae started to progress beyond the germ, they accumulated in the germ, causing loosening of the compacted starchy endosperm tissue (Figures 5Ai,B,C). However, when MoT hyphae progressed *via* seed coat rather than germ (Figure 5 Aii), the caryopsis endosperm were less damaged by MoT, and cell wall degradation started from the infection site (Figure 5D).

In Milan, MoT hyphae were mainly detected in the caryopsis coat tissues and in the germ part, with a prevalence of the germ region (Figures 5H,I). Due to the low infection rate in Milan, MoT hyphae only very slowly progressed toward the innermost endosperm (Figure 5).

### 3.6. Vertical seed-to-seed transmission of MoT

**Figure 6:**
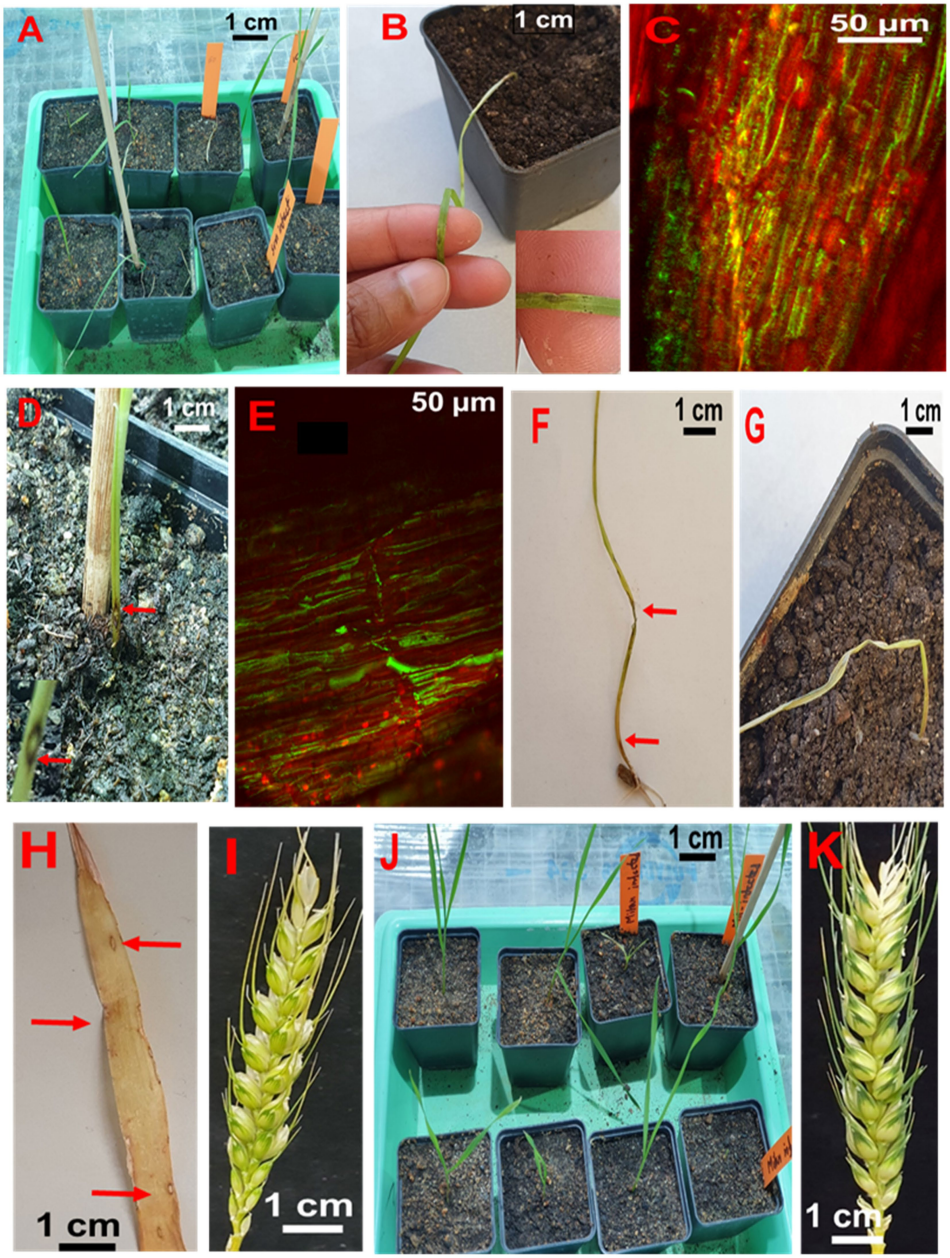
Testing seed-to-seed transmission of MoT. **(A)** Seedlings from infected Sumai 3 seeds in the climate chamber. **(B)** Characteristic eye-shaped blast symptoms on the first leaf of a Sumai 3 seedling (GS 11). **(C)** Fungal hyphae in infected Sumai 3 seedling leaf tissue visualized through CLSM. **(D)** Typical eye-shaped blast symptom on the stem of Sumai 3 (GS 11). **(E)** Longitudinal section of infected Sumai 3 stem displaying fungal growth in infected stem tissue by CLSM (GS 11). **(F)** Severely infected Sumai 3 seedling developed from MoT infected seed. **(G)** Sumai 3 seedling killed through MoT infection (GS 14). **(H)** Characteristic blast symptoms on older leaves of Sumai 3 (GS 21). **(I)** Asymptomatic ear from infected seeds of Sumai 3 (GS 65). **(J)** Seedlings from infected seeds of Milan (GS 11). **(K)** Asymptomatic ear from infected Milan seeds (GS 65).

**Figure 7:**
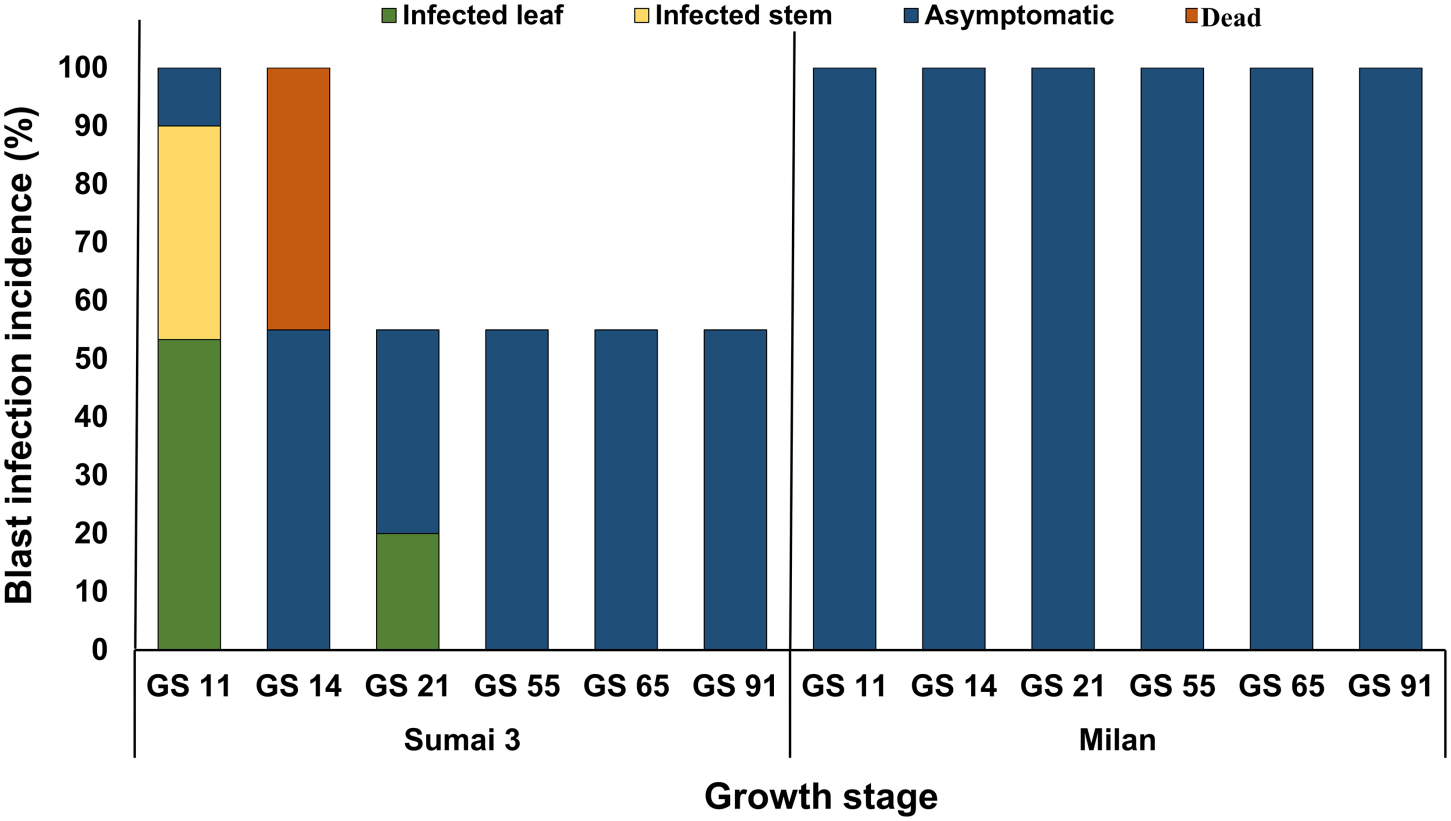
MoT infection incidence (%) in wheat plants grown from MoT infected seeds of Sumai 3 and Milan in the climate chamber. Sampling was performed at GS 11 (first leaf emerged), GS 14 (emergence of the 4th leaves), GS 21 (main stem with one tiller), GS 55 (half of the ear emerged above flag leaf ligule), GS 65 (mid flowering), and GS 91 (grain hard).

About half of the seedlings from infected seeds obtained from Sumai 3 ears inoculated at different maturity stages in the greenhouse showed symptoms on seedling leaves. At GS 11 (first leaf emerged), Sumai 3 seedlings showed symptoms on their first leaves (Figures 6A,B), about 36% of plants had characteristic blast symptoms on seedling stems (Figure 6D), and 10% were asymptomatic. Symptomatic leaves and stems were randomly collected, and MoT was reisolated from all infected samples. Interestingly, disease symptoms on ears were absent (Figures 6I,K). Due to severe blast severity, 45% of Sumai 3 seedlings were dead at GS 14 (emergence of the 4th leaves) (Figures 6G, 7).

At GS 21 (main stem with one tiller), 20% of seedlings showed blast symptoms on lower leaves (Figure 6H), and the rest were entirely asymptomatic. Surprisingly, after GS 21, no blast symptoms were observed in any parts of Sumai 3 plants, and MoT was also not detected by qPCR.

No visual blast symptoms were recorded in Milan from seedling to maturity stages, and no MoT was detected by CLSM, plating, and qPCR techniques (Figures 6, 7).

## 4. Discussion

Seeds are the primary units for crop production but may also play a key role in transmitting pathogens within a field or from one region to another. Neither the systemic growth of MoT from seed to seed nor the potential spread of MoT by seeds at the local, regional or long distance scale have been so far explored in-depth. In a previous study MoT could not be detected by reisolation from infected wheat plants (susceptible cultivar Apogee) later than 42 days after sowing (Martinez et al., 2021). In addition, till today, the role of cultivar resistance in the transmission and dissemination of MoT and its localization in seeds is not known.

The present study, conducted under controlled greenhouse and climate chamber conditions confirmed that MoT was able to establish in seeds after inoculation of ears at different flowering stages but there was no systemic (vertical) transmission detected to the next generation seeds (seed-to-seed). This was ensured through manually removing all infected leaves and dead plant parts in our trials immediately after observing any signs or symptoms of disease. The reason was to avoid cross-contamination of MoT from infected leaves or seedlings to ears or healthy plants. However, on plants grown from seeds of a susceptible cultivar, the pathogen caused symptoms on leaves and stems up to GS 21 from where it was reisolated indicating the potential of a local (horizontal) spread in the field originating from sowing infected seeds. In contrast, no such local spread of MoT was detectable in any stages of the resistant cultivar Milan.

Blast disease at ear maturity stages may have a significant economic impact on wheat production by producing a higher amount of abnormally formed seeds. In our greenhouse experiments, at all MoT inoculation time points, seed infection was almost 100% in Sumai 3 and 20–25% in Milan, resulting in poor quality grains. Previous studies on effects of MoT infection in wheat have demonstrated a positive correlation between MoT infection and grain quality (Goulart and Paiva, 2000; Islam et al., 2016; Singh et al., 2016; Surovy et al., 2020). Higher seed infection caused malformed seeds with lower protein content (Supplementary Figure S2). The grain protein content is essential for germination, and endosperm protein increases the seed water and oxygen uptake capacity. It also triggers faster germination and larger seedlings with high dry matter contents (Lopez and Grabe, 1973; Bulisani and Warner, 1980). Infection by *F. graminearum* (Nightingale et al., 1999; Prange et al., 2005; Gärtner et al., 2007; Arata et al., 2022), stripe rust (Devadas et al., 2014; Rozo-Ortega et al., 2021), leaf rust, septoria blotch (Castro et al., 2018), and tan spot (Fleitas et al., 2018) in wheat may also reduce grain protein content like MoT. Contrary to our results, some studies also showed that MoT-infected seeds contained higher protein content than healthy seeds (Urashima et al., 2009; Martínez et al., 2019).

When MoT conidia are spray inoculated on wheat ears, conidia start to germinate and infect the earlets (Ha et al., 2016). After infection at early ear emergence stage, MoT was found in husks and rachis, progressing toward the rachilla, colonizing the caryopsis germ region, and spreading in the endosperm. MoT present in the seed coat further progressed toward endosperm *via* penetrating the aleuric layer. Progression of MoT inside the wheat caryopsis was slower in Milan than in Sumai 3. Through CLSM, Ha et al. (2016) showed that disease progression was faster through the ear rachilla than palea in a susceptible cultivar.

The seed germ region plays a crucial role in reproducing new wheat plants. Most blast-infected seeds were tiny, low in weight, deformed, or showed black discoloration of their germ region. In the MoT sporulation assay with seeds, we found that most MoT conidia germinated from the germ region. Commonly, germs are a natural sink for essential amino acids, vitamins, fatty acids, minerals, phytosterols, and tocopherols (Zia Sherrell, 2021). As a result, MoT may prefer to colonize the wheat germ in order to get access to and utilize these nutrients. Our study confirmed that the MoT-infected wheat germ region is a preferred tissue where MoT can survive and sporulate for further dissemination. Rice blast-infected seeds also preferentially colonized the germ region (Manandhar et al., 1998; Long et al., 2001). Additionally, seeds from MoT-infected ears of both Sumai 3 and Milan produced MoT conidia which can serve as primary inoculum to spread blast disease into new areas or within a field after sowing. This is in partial contrast to previous reports explaining that only seeds from infected ears of susceptible cultivars but not from resistant cultivars may be a source of inoculum (Bernaux and Berti, 1981; Guerber and TeBeest, 2006).

The seed CLSM analyses revealed that MoT colonized the Sumai 3 seed germ region significantly stronger than in Milan. The localization of MoT inside the seeds was more difficult to track compared to leaves or stems. Producing thin seed sections of mature seeds with the microtome was quite challenging and paraffin fixation (Höch et al., 2021) followed by repeated washing caused damage to seed sections. Therefore, instead of paraffin fixation and sectioning with a microtome, we used razor blades (Wilkinson sword, Germany) for manual sectioning of wheat seeds. The AF and PI double staining easily distinguished plant tissue and fungal hyphae, after optimization of the staining time of AF (2 h) for seed samples. This double staining method is an effective and reliable method to visualize the presence of MoT hyphae inside the seed. AF is a specific chitin-binding dye and directly binds with the fungal cell wall. On the other hand, PI is specific for staining the nucleic acids. The concentration of AF was higher than of PI, as higher concentration of AF inhibits the binding of PI to fungal cells. As a result, AF stained fungal cells green and PI stained plant cells red. The CLSM examinations allowed us to analyze the presence of MoT hyphae within seeds, even in thicker cross-sectioned samples. Double staining with AF and PI has been previously used to localize *Fusarium* spp. in wheat, barley, and rye seeds (Jansen et al., 2005; Jin et al., 2021).

When MoT infected seeds were sown in the greenhouse, characteristic blast symptoms were observed at GS 11in younger leaves and stems of Sumai 3. The presence of MoT in infected leaves and stems was confirmed by observing the presence of conidia in infected parts by microscopy and agar plating methods. Severely infected seedlings died in later stages. Interestingly, after GS 21, no visual symptoms were observed in Sumai 3 plants, while no symptoms at all were noted on seedlings or ears derived from blast-infected Milan seeds. The disease incidence on seedlings of Sumai 3 grown from infected seeds ranged up to 64%, causing necrosis and death of seedlings. In contrast, Martinez et al. (2021) did not detect blast symptoms 41 days after sowing infected wheat seeds. Cruz et al. (2015) stated that old basal leaves are the initial inoculum source of wheat blast, and sampling at anthesis from the lower three senescent leaves resulted in recovery of MoT conidia. In their study, only four different susceptible cultivars were used and high numbers of MoT conidia were recovered from the lower basal leaves of all cultivars. However, in our study, we also used a resistant cultivar in order to explore the variability in symptom development and conidia production in cultivars differing in resistance. Our studies demonstrate, that on a resistant cultivar, sporulation may occur on infected seeds but is lacking on seedlings and adult plants grown from such seeds.

This is the first in-depth systematic report on the potential role of MoT seed infection in the short and long-distance dissemination of MoT. Transmission of seed-borne pathogens may depend on plant age (Giorcelli et al., 1996), cultivar (Roderick and Clifford, 1995), plant nutritional status (Chowdhury et al., 2022), and plant growing conditions (Nopsa and Pfender, 2014).

The plumule and radicle emerge by rupturing the germ coat. During emergence and cell differentiation, MoT conidia transmitted to plumule and radicle at the early cell differentiation stages may result in transfer of MoT from seed to seedling (Baker and Smith, 1966). There are two possibilities to transmit MoT from seed to wheat plants, first, by vertical transmission of MoT from seed to seed in a systemic manner, second, by horizontal transmission by MoT conidia by rain or wind. In our case, we only found conidia in early stages but not in ears. So, a vertical transmission of MoT can be excluded. In our system conidia either formed on infected seeds or on leaves and stems of young plants. They may be the primary inoculum spreading the disease within a field and finally causing ear infection. During cultural management in the field, by rain or wind, MoT conidia from seeds, infected seedlings, or leaves may spread to new seedlings or healthy ears of neighboring plants. This may ultimately incite an epidemic resulting in ear infection without systemic transfer of the pathogen.

Plating is an easy and handy method, however, with a high chance of low detection rate and false negative results (Hariharan and Prasannath, 2021). On the other hand, slight damage to living cells or tissues may hamper the detection by CLSM (DeVree et al., 2021). An optimized qPCR with species-specific primers thus appears a more reliable and sensitive technique to detect and trace minimal amounts of fungal biomass *in situ* (Zheng et al., 2019). If MoT was transmitted vertically from seed to ear in an asymptomatic manner, even low levels of fungal biomass in wheat stems and ears should be traceable by qPCR. However, by qPCR analysis no MoT DNA was detected in Milan (from seedling to ear) and Sumai 3 (after GS 21). This is the first comprehensive study combining qPCR, microscopy, and plating methods to precisely trace MoT colonization in wheat in order to unveil a potential transmission of MoT from seed to seed.

The lower infection and sporulation rates of MoT in Milan indicate that breeding for MoT-resistant cultivars is an excellent strategy to reduce the risk of MoT epidemics. In addition, the current study provides evidence that safeguarding healthy seeds to stop seed transmission appears an equally important strategy. The effectiveness of seed treatment with chemicals or biologicals therefore needs to be improved to avoid horizontal dissemination from infected seeds. The results from the present study are particularly important for wheat-producing regions where MoT has not been recorded thus far.

## Data availability statement

The raw data supporting the conclusions of this article will be made available by the authors, without undue reservation.

## Author contributions

MS and AT conceptualization and funding. MS: investigation, data analysis, visualization, and writing-original draft preparation. AT: project administration, writing, reviewing, and editing. TI: contributed to the basic design and provided a wheat blast isolate for the study. All authors read and approved the final version of the manuscript.

## Funding

The German academic exchange service (DAAD) funded MS for pursuing her Ph.D. degree at the Division of Plant Pathology and Crop Protection, Georg-August-Universität Göttingen, Germany. Lab consumables and Open Access publication fees were provided by the Division of Plant Pathology and Crop Protection, and the University Library (SUB) of the Georg-August-Universität Göttingen, Germany.

## Conflict of interest

The authors declare that the research was conducted in the absence of any commercial or financial relationships that could be construed as a potential conflict of interest.

## Publisher’s note

All claims expressed in this article are solely those of the authors and do not necessarily represent those of their affiliated organizations, or those of the publisher, the editors and the reviewers. Any product that may be evaluated in this article, or claim that may be made by its manufacturer, is not guaranteed or endorsed by the publisher.

## Supplementary material

The Supplementary material for this article van be found online at: https://www.frontiersin.org/articles/10.3389/fmicb.2023.1040605/full#supplementary-material

## References

[ref1] Alisaac E. Rathgeb A. Karlovsky P. Mahlein A.-K. (2020). Fusarium head blight: effect of infection timing on spread of *Fusarium graminearum* and spatial distribution of deoxynivalenol within wheat spikes. Microorganisms 9:79. doi: 10.3390/microorganisms9010079, PMID: 33396894 PMC7823776

[ref2] Arata G. J. Martínez M. Elguezábal C. Rojas D. Cristos D. Dinolfo M. I.. (2022). Effects of sowing date, nitrogen fertilization, and *Fusarium graminearum* in an Argentinean bread wheat: integrated analysis of disease parameters, mycotoxin contamination, grain quality, and seed deterioration. J. Food Compost. Anal. 107:104364. doi: 10.1016/j.jfca.2021.104364

[ref3] Baker K. F. Smith S. H. (1966). Dynamics of seed transmission of plant pathogens. Annu. Rev. Phytopathol. 4, 311–332. doi: 10.1146/annurev.py.04.090166.001523

[ref4] Bernaux P. Berti G. (1981). Évolution de la sensibilité des glumelles du riz à *Pyricularia oryzae* Cav. et à *Drechslera oryzae* (Br. de Haan) Sub. et Jain: conséquences pour la transmission des maladies. Agronomie 1, 261–264. doi: 10.1051/agro:19810402

[ref5] Bulisani E. A. Warner R. L. (1980). Seed protein and nitrogen effects upon seedling vigor in wheat. Agron. J. 72, 657–662. doi: 10.2134/agronj1980.00021962007200040021x

[ref6] Castro A. C. Fleitas M. C. Schierenbeck M. Gerard G. S. Simόn M. R. (2018). Evaluation of different fungicides and nitrogen rates on grain yield and bread-making quality in wheat affected by Septoria tritici blotch and yellow spot. J. Cereal Sci. 83, 49–57. doi: 10.1016/j.jcs.2018.07.014

[ref7] Ceresini P. C. Castroagudín V. L. Rodrigues F. Á. Rios J. A. Aucique-Pérez C. E. Moreira S. I.. (2019). Wheat blast: from its origins in South America to its emergence as a global threat: wheat blast. Mol. Plant Pathol. 20, 155–172. doi: 10.1111/mpp.12747, PMID: 30187616 PMC6637873

[ref8] Ceresini P. C. Castroagudín V. L. Rodrigues F. Á. Rios J. A. Eduardo Aucique-Pérez C. Moreira S. I.. (2018). Wheat blast: past, present, and future. Annu. Rev. Phytopathol. 56, 427–456. doi: 10.1146/annurev-phyto-080417-050036, PMID: 29975608

[ref9] Chowdhury M. S. R. Rahman M. A. Nahar K. Dastogeer K. M. G. Hamim I. Mohiuddin K. M. (2022). Mineral nutrient content of infected plants and allied soils provide insight into wheat blast epidemics. Heliyon 8:e08966. doi: 10.1016/j.heliyon.2022.e08966, PMID: 35243086 PMC8873539

[ref10] Cruz C. D. Kiyuna J. Bockus W. W. Todd T. C. Stack J. P. Valent B. (2015). *Magnaporthe oryzae* conidia on basal wheat leaves as a potential source of wheat blast inoculum. Plant Pathol. 64, 1491–1498. doi: 10.1111/ppa.12414

[ref11] Cruz C. D. Valent B. (2017). Wheat blast disease: danger on the move. Trop. Plant Pathol. 42, 210–222. doi: 10.1007/s40858-017-0159-z

[ref12] Devadas R. Simpfendorfer S. Backhouse D. Lamb D. W. (2014). Effect of stripe rust on the yield response of wheat to nitrogen. Crop J. 2, 201–206. doi: 10.1016/j.cj.2014.05.002

[ref13] DeVree B. T. Steiner L. M. Glazowska S. Ruhnow F. Herburger K. Persson S.. (2021). Current and future advances in fluorescence-based visualization of plant cell wall components and cell wall biosynthetic mechineries. Biotechnol. Biofuels 14:78. doi: 10.1186/s13068-021-01922-0, PMID: 33781321 PMC8008654

[ref14] Elmer W. H. (2001). Seeds as vehicles for pathogen importation. Biol. Invasions 3, 263–271. doi: 10.1023/A:1015217308477

[ref15] Faivre-Rampant O. Geniès L. Piffanelli P. Tharreau D. (2013). Transmission of rice blast from seeds to adult plants in a non-systemic way. Plant Pathol. 62, 879–887. doi: 10.1111/ppa.12003

[ref17] Fleitas M. C. Schierenbeck M. Gerard G. S. Dietz J. I. Golik S. I. Simόn M. R. (2018). Breadmaking quality and yield response to the green leaf area dration caused by fluxapyroxad under three nitrogen rates in wheat affected with tan spot. Crop Prot. 106, 201–209. doi: 10.1016/j.cropprto.2018.01.004

[ref18] Food and Agriculture Organization of the United Nations (2015). FAO Statistical Pocketbook 2015: World Food and Agriculture. Rome: Food and Agriculture Organization of the United Nations.

[ref19] Gärtner B. H. Munich M. Kleijer G. Mascher F. (2007). Characterisation of kernel resistance against Fusarium infection in spring wheat by baking quality and mycotoxin assessments. Eur. J. Plant Pathol. 120, 61–68. doi: 10.1007/s10658-007-9198-5

[ref20] Giorcelli A. Vietto L. Anselmi N. Gennaro M. (1996). Influence of clonal susceptibility, leaf age and inoculum density on infections by *Melampsora larici-Populina* races E1 and E3. Eur. J. Plant Pathol. 26, 323–331. doi: 10.1111/j.1439-0329.1996.tb01078.x

[ref21] Goulart A. C. P. Paiva F. A. (2000). Wheat yield losses due to *Pyricularia grisea*, in 1991 and 1992, in the state of Mato Grosso do Sul. Summa Phytopathol. 26, 279–282.

[ref22] Goulart A. C. P. Paiva F. A. De Mesquita A. N. (1990). *Pyricularia oryzae* in wheat seeds: incidence, transmission and survival. Annu. Wheat Newsl. 36, 49–50.

[ref23] Guerber C. TeBeest D. O. (2006). Infection of rice seed grown in Arkansas by *Pyricularia grisea* and transmission to seedlings in the field. Plant Dis. 90, 170–176. doi: 10.1094/PD-90-0170, PMID: 30786408

[ref24] Gupta D. R. Khanom S. Rohman M. M. Hasanuzzaman M. Surovy M. Z. Mahmud N. U.. (2021). Hydrogen peroxide detoxifying enzymes show different activity patterns in host and non-host plant interactions with *Magnaporthe oryzae Triticum* pathotype. Physiol. Mol. Biol. Plants 27, 2127–2139. doi: 10.1007/s12298-021-01057-4, PMID: 34629783 PMC8484409

[ref25] Gupta D. R. Surovy M. Z. Mahmud N. U. Chakraborty M. Paul S. K. Hossain M. S.. (2020). Suitable methods for isolation, culture, storage and identification of wheat blast fungus *Magnaporthe oryzae Triticum* Pathotype. Phytopathol. Res. 2:30. doi: 10.1186/s42483-020-00070-x

[ref26] Ha X. Koopmann B. von Tiedemann A. (2016). Wheat blast and fusarium head blight display contrasting interaction patterns on ears of wheat genotypes differing in resistance. Phytopathology 106, 270–281. doi: 10.1094/PHYTO-09-15-0202-R, PMID: 26574785

[ref27] Hariharan G. Prasannath K. (2021). Recent advances in molecular diagnostics of fungal plant pathogens: a mini review. Front. Cell. Infect. Microbiol. 10:600234. doi: 10.3389/fcimb.2020.600234, PMID: 33505921 PMC7829251

[ref28] Höch K. Koopmann B. von Tiedemann A. (2021). Lignin composition and timing of cell wall lignification are involved in *Brassica napus* resistance to stem rot caused by *Sclerotinia sclerotiorum*. Phytopathology 111, 1438–1448. doi: 10.1094/PHYTO-09-20-0425-R, PMID: 33386067

[ref29] Igarashi S. Utiamada C. M. Igarashi L. C. Kazuma A. H. Lopes R. S. (1985). Pyricularia em trigo. 1. ocorrência de *Pyricularia* sp. no estado do Paraná. Fitopatol. Bras. 11, 351–352.

[ref30] Islam M. T. Croll D. Gladieux P. Soanes D. M. Persoons A. Bhattacharjee P.. (2016). Emergence of wheat blast in Bangladesh was caused by a south American lineage of *Magnaporthe oryzae*. BMC Biol. 14:84. doi: 10.1186/s12915-016-0309-7, PMID: 27716181 PMC5047043

[ref31] Islam M. T. Gupta D. R. Hossain A. Roy K. K. He X. Kabir M. R.. (2020). Wheat blast: a new threat to food security. Phytopathol. Res. 2:28. doi: 10.1186/s42483-020-00067-6

[ref32] Islam M. T. Kim K.-H. Choi J. (2019). Wheat blast in Bangladesh: the current situation and future impacts. Plant Pathol. J. 35, 1–10. doi: 10.5423/PPJ.RW.08.2018.0168, PMID: 30828274 PMC6385656

[ref33] Jansen C. von Wettstein D. Schafer W. Kogel K.-H. Felk A. Maier F. J. (2005). Infection patterns in barley and wheat spikes inoculated with wild-type and trichodiene synthase gene disrupted *Fusarium graminearum*. Proc. Natl. Acad. Sci. 102, 16892–16897. doi: 10.1073/pnas.0508467102, PMID: 16263921 PMC1283850

[ref34] Jin Z. Solanki S. Ameen G. Gross T. Poudel R. S. Borowicz P.. (2021). Expansion of internal hyphal growth in fusarium head blight–infected grains contributes to the elevated mycotoxin production during the malting process. Mol. Plant Microbe Interact. 34, 793–802. doi: 10.1094/MPMI-01-21-0024-R, PMID: 33720745

[ref35] Long D. H. Correll J. C. Lee F. N. TeBeest D. O. (2001). Rice blast epidemics initiated by infested rice grain on the soil surface. Plant Dis. 85, 612–616. doi: 10.1094/PDIS.2001.85.6.612, PMID: 30823027

[ref36] Lopez A. Grabe D. F. (1973). Effect of protein content on seed performance in wheat (*Triticum aestivum* L.). in Proceedings of the Association of Official Seed Analysts (Association of Official Seed Analysts and the Society of Commercial Seed Technologists (SCST), 106–116.

[ref37] Maciel J. L. N. (2018). “Diseases affecting wheat: wheat blast” in Burleigh Dodds Series in Agricultural Science. ed. Oliver R. (London: Burleigh Dodds Science Publishing), 155–169. doi: 10.19103/AS.2018.0039.08

[ref38] Maciel J. L. N. Ceresini P. C. Castroagudin V. L. Zala M. Kema G. H. J. McDonald B. A. (2014). Population structure and pathotype diversity of the wheat blast pathogen *Magnaporthe oryzae* 25 years after its emergence in Brazil. Phytopathology 104, 95–107. doi: 10.1094/PHYTO-11-12-0294-R, PMID: 23901831

[ref39] Manandhar H. K. Jorgensen H. J. L. Smedegaard-Petersen V. Mathur S. B. (1998). Seedborne infection of rice by *Pyricularia oryzae* and its transmission to seedlings. Plant Dis. 82, 1093–1099. doi: 10.1094/PDIS.1998.82.10.1093, PMID: 30856768

[ref40] Martínez S. I. Sanabria A. Fleitas M. C. Consolo V. F. Perelló A. (2019). Wheat blast: aggressiveness of isolates of *Pyricularia oryzae* and effect on grain quality. J. King Saud Univ. Sci. 31, 150–157. doi: 10.1016/j.jksus.2018.05.003

[ref41] Martinez S. I. Wegner A. Bohnert S. Schaffrath U. Perelló A. (2021). Tracing seed to seedling transmission of the wheat blast pathogen *Magnaporthe oryzae* pathotype *Triticum*. Plant Pathol. 70, 1562–1571. doi: 10.1111/ppa.13400

[ref42] McGee D. C. (1979). Epidemiological aspects of seed disease control. J. Seed Tech. 4, 96–98.

[ref43] Monsur M. Ahmed M. Haque A. Jahan Q. Ansari T. Latif M.. (2016). Cross infection between rice and wheat blast pathogen *Pyricularia oryzae*. Bangladesh Rice J. 20, 21–29. doi: 10.3329/brj.v20i2.34125

[ref44] Mottaleb K. A. Singh P. K. Sonder K. Kruseman G. Tiwari T. P. Barma N. C. D.. (2018). Threat of wheat blast to South Asia's food security: an ex-ante analysis. PLoS One 13:e0197555. doi: 10.1371/journal.pone.0197555, PMID: 29782528 PMC5962063

[ref45] Nallathambi P. Umamaheswari C. Sandeep K. L. Manjunatha C. Berliner J. (2021). “Mechanism of seed transmission and seed infection in major agricultural crops in India” in Seed-Borne Diseases of Agricultural Crops: Detection, Diagnosis and Management. eds. Kumar R. Gupta A. (Singapore: Springer Nature Singapore Pte Ltd. 2020), 749–791.

[ref46] Nightingale M. J. Marchylo B. A. Clear R. M. Dexter J. E. Preston K. R. (1999). Fusarium head blight: effect of fungal proteases on wheat storage proteins. Cereal Chem. 76, 150–158. doi: 10.1094/CCHEM.1999.76.1.150

[ref47] Nopsa J. F. H. Pfender W. F. (2014). A latent period duration model for wheat stem rust. Plant Dis. 98, 1358–1363. doi: 10.1094/PDIS-11-13-1128-RE, PMID: 30703926

[ref48] Paul S. K. Mahmud N. U. Gupta D. R. Rani K. Kang H. Wang G. L.. (2022). *Oryzae* pathotype of *Magnaporthe oryzae* can cause typical blast disease symptoms on both leaves and spikes of wheat under a growth room condition. Phytopathol. Res. 4:9. doi: 10.1186/s42483-022-00114-4

[ref49] Pizolotto C. A. Maciel J. L. N. Fernandes J. M. C. Boller W. (2019). Saprotrophic survival of *Magnaporthe oryzae* in infested wheat residues. Eur. J. Plant Pathol. 153, 327–339. doi: 10.1007/s10658-018-1578-5

[ref50] Prange A. Birzele B. Krämer J. Meier A. Modrow H. Köhler P. (2005). Fusarium-inoculated wheat: deoxynivalenol contents and baking quality in relation to infection time. Food Control 16, 739–745. doi: 10.1016/j.foodcont.2004.06.013

[ref51] Raveloson H. Ratsimiala Ramonta I. Tharreau D. Sester M. (2018). Long-term survival of blast pathogen in infected rice residues as major source of primary inoculum in high altitude upland ecology. Plant Pathol. 67, 610–618. doi: 10.1111/ppa.12790

[ref52] Reis E. M. (1995). Sobrevivência de *Pyricularia oryzae*, associada as sementes de trigo. Summa Phytopathol. 21:43.

[ref53] Roderick H. W. Clifford B. C. (1995). Variation in adult plant resistance to powdery mildew in spring oats under field and laboratory conditions. Plant Pathol. 44, 366–373. doi: 10.1111/j.1365-3059.1995.tb02789.x

[ref54] Rozo-Ortega G. P. Serrago R. A. Lo Valvo P. J. Fleitas M. C. Simón M. R. Miralles D. J. (2021). Grain yield, milling and breadmaking quality responses to foliar diseases in old and modern Argentinean wheat cultivars. J. Cereal Sci. 99:103211. doi: 10.1016/j.jcs.2021.103211

[ref55] Shahzad R. Khan A. L. Bilal S. Asaf S. Lee I.-J. (2018). What is there in seeds? Vertically transmitted endophytic resources for sustainable inprovement in plant growth. Front. Plant Sci. 9:24. doi: 10.3389/fpls.2018.00024, PMID: 29410675 PMC5787091

[ref56] Shiferaw B. Smale M. Braun H.-J. Duveiller E. Reynolds M. Muricho G. (2013). Crops that feed the world 10. Past successes and future challenges to the role played by wheat in global food security. Food Sec. 5, 291–317. doi: 10.1007/s12571-013-0263-y

[ref57] Shizhen W. Jiaoyu W. Zhen Z. Zhongna H. Xueming Z. Rongyao C.. (2021). The risk of wheat blast in rice–wheat co-planting regions in China: MoO strains of *Pyricularia oryzae* cause typical symptom and host reaction on both wheat leaves and spikes. Phytopathology 111, 1393–1400. doi: 10.1094/PHYTO-10-20-0470-R, PMID: 33471560

[ref58] Singh P. K. Gahtyari N. C. Roy C. Roy K. K. He X. Tembo B.. (2021). Wheat blast: a disease spreading by intercontinental jumps and its management strategies. Front. Plant Sci. 12:710707. doi: 10.3389/fpls.2021.710707, PMID: 34367228 PMC8343232

[ref59] Singh R. P. Singh P. K. Rutkoski J. Hodson D. P. He X. Jørgensen L. N.. (2016). Disease impact on wheat yield potential and prospects of genetic control. Annu. Rev. Phytopathol. 54, 303–322. doi: 10.1146/annurev-phyto-080615-095835, PMID: 27296137

[ref60] Stokstad E. (2007). Deadly wheat fungus threatens world's breadbaskets. Science 315, 1786–1787. doi: 10.1126/science.315.5820.1786, PMID: 17395806

[ref61] Surovy M. Z. Dutta S. Mahmud N. U. Gupta D. R. Farhana T. Paul S. K.. (2022). Probiotic *Bacillus* species: promising biological control agents for managing worrisome wheat blast disease. Preprints. doi: 10.20994/preprints202211.0382.v1

[ref62] Surovy M. Z. Mahmud N. U. Bhattacharjee P. Hossain M. S. Mehebub M. S. Rahman M.. (2020). Modulation of nutritional and biochemical properties of wheat grains infected by blast fungus *Magnaporthe oryzae Triticum* Pathotype. Front. Microbiol. 11:1174. doi: 10.3389/fmicb.2020.01174, PMID: 32714284 PMC7344263

[ref63] Téllez L. C. Chavez A. Estigarribia P. P. Reyes M. Cazal C. Heesacker A.. (2022). Caninde2/Milan: promising wheat line to discover novel genes for resistant to wheat blast. Crop Breed. Appl. Biotechnol. 22:e40032221. doi: 10.1590/1984-70332022v22n2a11

[ref64] Tembo B. Mulenga R. M. Sichilima S. M’siska K. K. Mwale M. Chikoti P. C.. (2020). Detection and characterization of fungus (*Magnaporthe oryzae* pathotype *Triticum*) causing wheat blast disease on rain-fed grown wheat (*Triticum aestivum* L.) in Zambia. PLoS One 15:e0238724. doi: 10.1371/journal.pone.0238724, PMID: 32956369 PMC7505438

[ref65] Tesfaye K. (2021). Climate change in the hottest wheat regions. Nat. Food 2, 8–9. doi: 10.1038/s43016-020-00218-0 37117660

[ref66] Tingting W. (2014). Epidemiology, phytopathological and molecular differentiation and leaf infection process of diverse strains of *Magnaporthe* spp. on wheat and rice. Ph. D. dissertation. Göttingen: Georg-August-Universität Göttingen.

[ref67] Urashima A. S. Grosso C. R. F. Stabili A. Freitas E. G. Silva C. P. Netto D. C. S.. (2009). “Effect of *Magnaporthe grisea* on seed germination, yield and quality of wheat” in Advances in Genetics, Genomics and Control of Rice Blast Disease. eds. Wang G. L. Valent B. (Dordrecht: Springer), 267–277. doi: 10.1007/978-1-4020-9500-9_27

[ref68] Urashima A. S. Lavorent N. A. Goulart A. C. P. Mehta Y. R. (2004). Resistance spectra of wheat cultivars and virulence diversity of *Magnaporthe grisea* isolates in Brazil. Fitopatol. Bras. 29, 511–518. doi: 10.1590/S0100-41582004000500007

[ref69] Urashima A. S. Leite S. F. Galbieri R. (2007). Eficiência da disseminação aérea em *Pyricularia grisea*. Summa Phytopathol. 33, 275–279. doi: 10.1590/S0100-54052007000300011

[ref70] Winston J. (2015). Aggressive Plant Fungus Threatens Wheat Production. Available a: https://www.biomedcentral.com/about/press-centre/science-press-releases/27-02-2015 ().

[ref71] Zadoks J. C. Chang T. T. Konzak C. F. (1974). A decimal code for the growth stages of cereals. Weed Res. 14, 415–421. doi: 10.1111/j.1365-3180.1974.tb01084.x

[ref72] Zheng X. Lopisso D. T. Eseola A. B. Koopmann B. Tiedemann A. (2019). Potential for seed transmission of *Verticillium longisporum* in oilseed rape (*Brassica napus*). Plant Dis. 103, 1843–1849. doi: 10.1094/PDIS-11-18-2024-RE, PMID: 31124750

[ref73] Zia Sherrell M. (2021). Look out, wheaties — wheat germ might be the new breakfast of health champs. Nutrition

