# Supplementary material for: Role of seed infection for the near and far distance dissemination of wheat blast caused by Magnaporthe oryzae pathotype Triticum
Source: Front Microbiol. 2023 Feb 1;14:1040605. doi: 10.3389/fmicb.2023.1040605 (PMC9929367; doi:10.3389/fmicb.2023.1040605)
Supplement: Supplementary file 1 [file Data_Sheet_1.pdf]

Supplementary Material

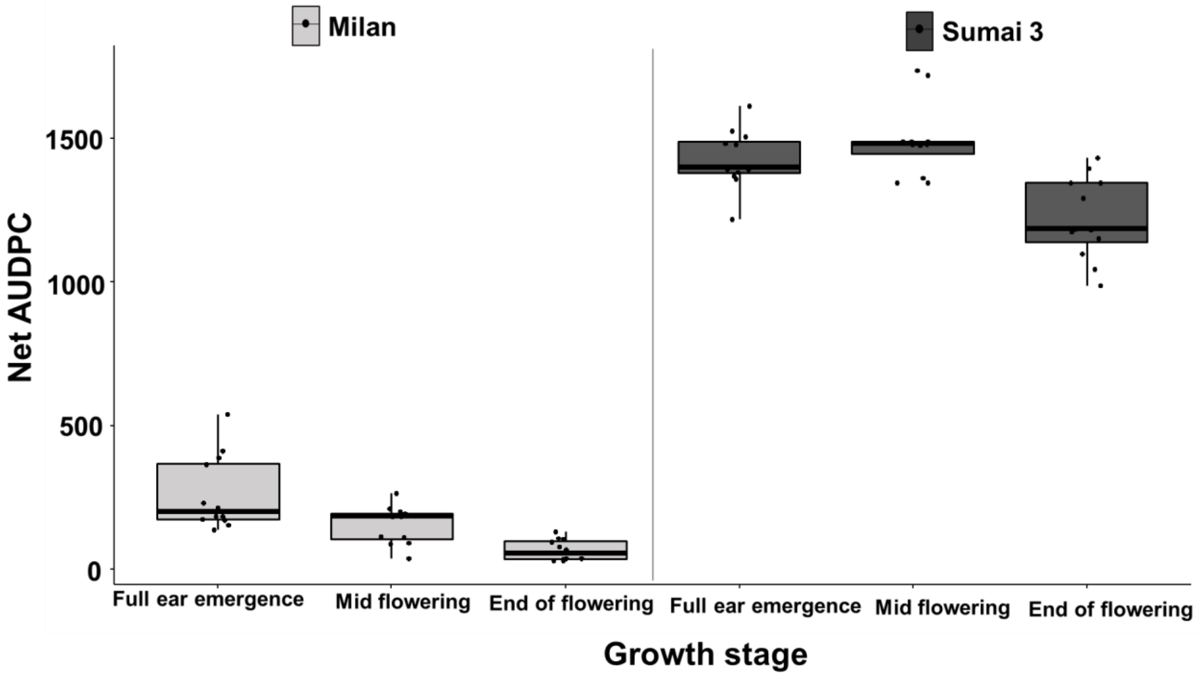

Supplementary figure 1: The net area under disease progressive curve (AUDPC) of Sumai 3 and Milan ears inoculated with MoT at different ear maturity time points. The wheat ears were spray inoculated with a conidial suspension of 1×10^5 conidia/ml at full ear emergence GS 59, mid flowering GS 65, and end of flowering GS 69 stages. n = 12; single replication contained 2 ears of each cultivar at each inoculation time point; $p = 0.05$. Each data point represents one replicate consisting of 2 ears per pot.

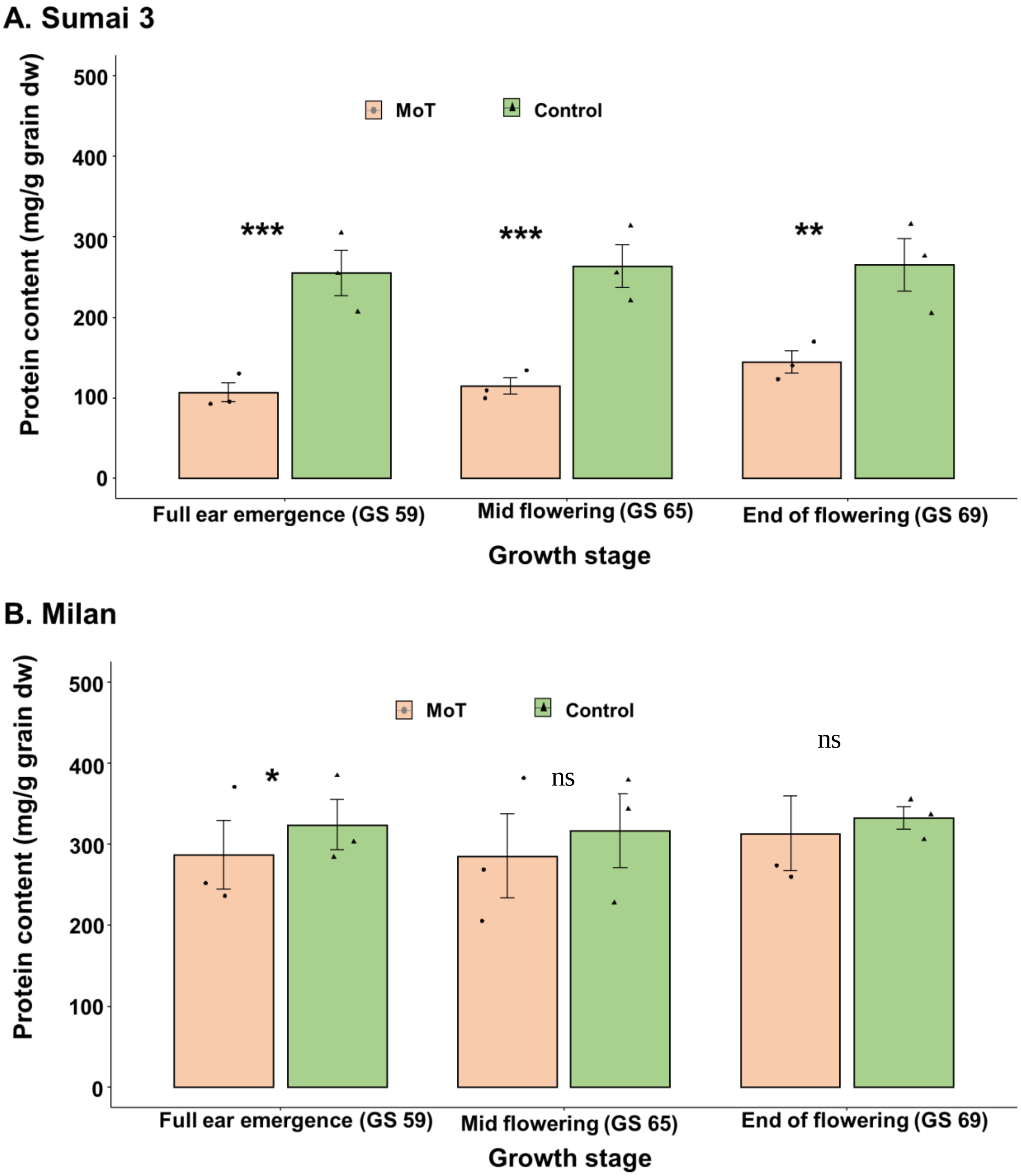

Supplementary figure 2: Effect of different inoculation time points of MoT on grain protein content (mg/g) of Sumai 3 (A) and Milan (B) cultivar (n = 3 and single replication contained eight pooled ear samples) in greenhouse conditions. The wheat ears were spray inoculated with a conidial suspension of 1×10^5 conidia/ml at full ear emergence GS 59, mid flowering GS 65, and end of flowering GS 69 stages. Paired t-test was performed (n = 3; one replication contained 30 seeds of each cultivar at each inoculation time point; *p* = 0.05). Each data point represents one replicate consisting of 8 ears from 4 pots. *** p≤0.001; ** p≤0.01; * p≤0.05 ; ns p>0.05.
